# Correction: Prevalence of AI-Supported Sexual Activities Among Adults in Germany: Results from a National Online Survey

**DOI:** 10.1007/s10508-026-03612-0

**Published:** 2026-08-05

**Authors:** Nicola Döring, Veronika Mikhailova, M. Rohangis Mohseni

**Affiliations:** Department of Economic Sciences and Media, Technische Universität Ilmenau, Ehrenbergstr. 29, 98693 Ilmenau, Germany

**Correction to: Archives of Sexual Behavior** **https://doi.org/10.1007/s10508-025-03382-1**

Due to an error during production, the heading "% [CI]" was missing in a number of places from Tables 2–6 in the article as originally published.

The original article has been corrected.

